# Supplementary figures and images for: 3D ophthalmic ultrasonography at the slit lamp using existing ultrasound systems
Source: PLoS One. 2025 Jan 28;20(1):e0317885. doi: 10.1371/journal.pone.0317885 (PMC11774342; doi:10.1371/journal.pone.0317885)

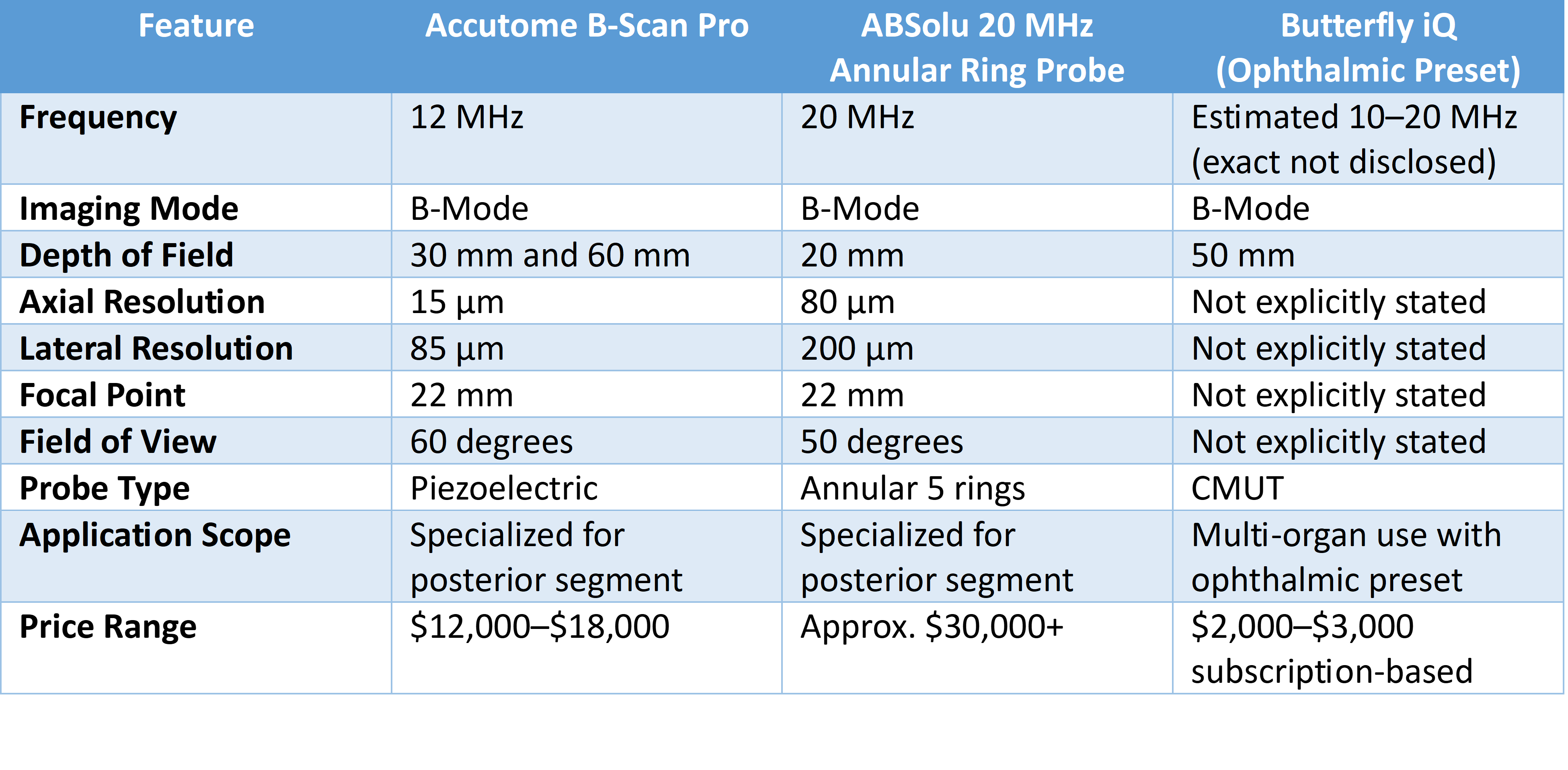

Supplement: S1 Table — (TIF) [file pone.0317885.s001.tif]

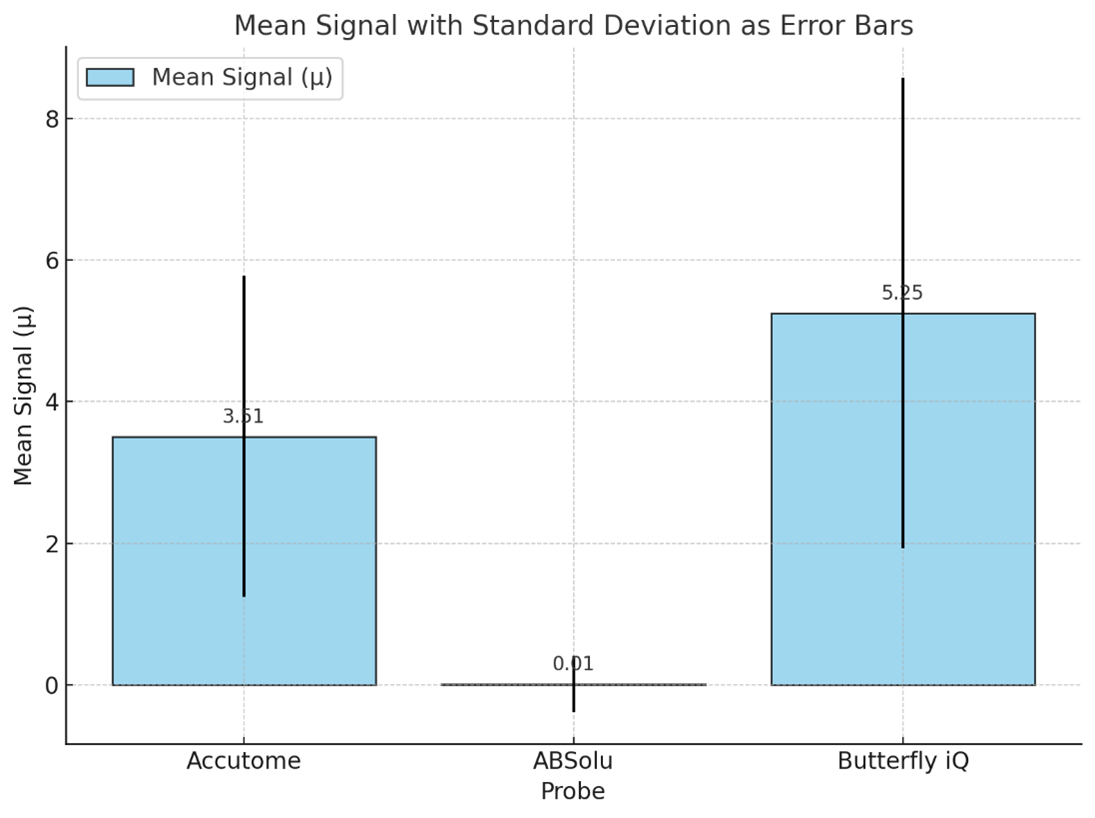

Supplement: S1 Fig — Mean Signal (μ) is labelled in blue with Standard Deviation (σ, Noise) as error bars. (TIFF) [file pone.0317885.s002.tiff]
