# Supplementary material for: 3D ophthalmic ultrasonography at the slit lamp using existing ultrasound systems
Source: PLoS One. 2025 Jan 28;20(1):e0317885. doi: 10.1371/journal.pone.0317885 (PMC11774342; doi:10.1371/journal.pone.0317885)
Supplement: Attachment — Submitted filename: Response to Reviewers.docx [file pone.0317885.s003.docx]

**Response to Reviewers**

We are enclosing a revised version of our manuscript entitled “3D ophthalmic ultrasonography at the slit lamp using existing ultrasound systems”. The reviewers’ comments are in blue text. Author responses are in black text.

We would like to thank the reviewers for their comments and efforts in improving our manuscript. Our revised manuscript addresses all comments made by the reviewers. A detailed, point-by-point reply to each of the comments is listed below.

Review Comments to the Author:

Reviewer #1: In the manuscript “3D ophthalmic ultrasonography at the slit lamp using existing ultrasound systems” the authors propose an adapted slit lamp for acquiring 3D ultrasonic images of the eye.

The main concern is that, for my understanding, the same propose has been previously published in: <https://iovs.arvojournals.org/article.aspx?articleid=2791001> and this new manuscript lacks originality.

**Response**: Thank you, Reviewer #1, for your attention to similar publications in this field. We appreciate the reference to the conference abstract, which highlights an earlier approach to 3D ocular ultrasound. However, we would like to clarify that this referenced abstract represents a conference poster presentation, not a formal, peer-reviewed manuscript.

While both approaches aim to adapt 3D ultrasound for ophthalmic use, our manuscript presents original contributions by using different ultrasound devices—specifically, the Accutome B-Scan Pro® (12 MHz probe), Quantel ABSolu® (20 MHz annular probe), and Butterfly iQ®—compared to the Ellex Eye Cubed (10 MHz) used in the referenced abstract. Additionally, our work features unique 3D reconstructions of both phantoms and pathological conditions, which were not included in the abstract. These distinctions highlight the novelty and expanded scope of our study.

Moreover, the authors argue that previous 3D ophthalmic US scans were not popular in clinical practice and propose a 3D ophthalmic system coupled to a slit lamp as a solution. I’m not sure about the advantages of having the US device coupled to a slit lamp since the two imaging approaches can’t be implemented at the same time.

**Response**: Thank you for your thoughtful comments. We agree that simultaneous use of ultrasound and slit lamp biomicroscopy was not intended or proposed in our manuscript.

The purpose of integrating the ultrasound device with a slit lamp is to leverage the stability and precision that a slit lamp provides, thereby addressing several limitations associated with traditional handheld ultrasound. Specifically:

1. **Enhanced Accessibility**: By adapting the system to fit onto an existing slit lamp, any examination lane equipped with a slit lamp could potentially become capable of performing ultrasound, expanding access to 3D ultrasound capabilities in clinical settings.
2. **Increased Precision and Consistency**: Unlike manual ultrasound scanning, which is subject to variability due to user skill and technique, mounting the ultrasound probe on a slit lamp enables precise, automated movement along a predefined path. This reduces manual errors and allows more consistent and accurate 3D reconstruction of ocular anatomy.
3. **Improved Patient Stability**: The chin rest and head support of a slit lamp help maintain patient positioning, reducing motion artifacts and variability in image acquisition. This stability is particularly beneficial for producing high-quality 3D ultrasound volumes with minimal dependency on the operator’s expertise.
4. **Broadened Usability**: This system could make 3D ultrasound acquisition more accessible to technicians without specialized ultrasound training, similar to how fundus imaging is handled in many clinics. This would allow for more routine and efficient acquisition of ultrasound volumes.

Related to: “Institutional Review Board approval was obtained from the University of California, Irvine, for clinical data collection.” Isn’t a protocol number?

**Response**: Thank you for drawing attention to this oversight. The protocol number for IRB approval from the University of California, Irvine, is #20195254. This information will be included in the revised manuscript.

There is missed information about the image acquisition protocol. How is the probe coupled to the cornea? Do the patients need some preparation for the study? Some information is provided just at the end on the Discussion section, but is should be detailed in M&M.

**Response**: Thank you for highlighting this. The image acquisition protocol in the Methods section describes the procedure, stating: “Patients received Tetracaine Hydrochloride Ophthalmic Solution, USP 0.5% (Alcon, Geneva, Switzerland) eye drops, were positioned in a slit lamp, and GenTeal Gel (Alcon, Geneva, Switzerland) was applied to the US probe as a lubricating agent. The probe was brought into contact with patients' corneas, and horizontal scans were obtained.”

The comparison of the results obtained with different probes is not relevant (as Fig 4, Fig 5 and related text) since it is intrinsic to the characteristics of the commercial probes. Also, information about the probes is missed, like BW, f-number, focal distance, resolution, etc.

**Response**: The authors acknowledge that the results obtained with each ultrasound probe reflect the inherent characteristics of the respective commercial devices. However, the primary purpose of including multiple ultrasound probes in this study was to demonstrate the 3D ocular ultrasound device’s compatibility across different imaging systems and to process the native data from each to create 3D models of ocular anatomy.

To clarify any potential ambiguity about the ultrasound systems’ specifications, we have added the following to the Methods section: “The A12 and AQ20 probes acquired all data using default settings, while the BiQ was used exclusively with the ophthalmic preset function.” Detailed specifications, such as bandwidth, f-number, focal distance, and resolution, can be found on the manufacturers’ websites if needed.

The legend of Fig 7 is incomplete in the draft.

**Response**: We apologize for this oversight and have adjusted all figures and legends to align with PLOS ONE’s formatting guidelines.

Reviewer #2:
The work is genuine, however its application is very difficult and full of challenges.
This paper can be considered a platform to invent a new device, that needs lot of technology and engineering.
The photos are nice but not adding superior information to the usual US or OCT

**Response**: Thank you, Reviewer #2, for your insightful feedback. We agree that implementing the novel 3D ocular ultrasound device presents significant challenges. However, as you suggest, we hope that demonstrating its potential on phantoms, pathological cases, and healthy controls can reinvigorate research in this area, which has seen limited progress over the past 30 years.

We believe this approach could enhance traditional ultrasound imaging, particularly for visualizing complex intraocular structures, such as tractional retinal detachments. Although there will undoubtedly be substantial technological and engineering hurdles, we are optimistic that these can be overcome with continued diligence, perseverance, and enthusiasm.

While OCT and conventional ultrasound remain essential tools in ophthalmology, we hope that 3D ultrasound reconstruction can add value by providing enhanced spatial visualization and unique coronal views, especially for patients lacking an optical view to the posterior pole. Our goal is not to compete with OCT but to expand the capabilities of ultrasound in current clinical applications and potentially enable its use for examining periocular structures.

Reviewer #3:
Areas for Improvement:
Lack of Quantitative Analysis:
While the paper provides a qualitative evaluation of the 3D reconstructions, it lacks quantitative measurements or objective performance metrics for assessing the accuracy and resolution of the images produced by the different probes.
Including a detailed comparison of the image resolution, noise levels, and accuracy of the reconstructed 3D models would enhance the scientific rigor of the study and make the findings more robust.

**Response:** We thank Reviewer #3 for highlighting the importance of quantitative analysis to assess differences in imaging performance across systems. We acknowledge that incorporating quantitative metrics, such as image resolution, noise levels, and 3D model accuracy, could add further depth to this study. Indeed, comparisons of performance metrics across imaging systems have been widely explored in fields like Optical Coherence Tomography (OCT).

However, the aim of this manuscript is not to evaluate or compare the performance metrics of individual ultrasound systems. Rather, our focus is to demonstrate the feasibility of a custom 3D ocular ultrasound acquisition device that utilizes native data from various ultrasound systems to generate 3D reconstructions of ocular anatomy. It is important to note that our system operates independently of the inherent performance characteristics of any particular manufacturer’s ultrasound equipment.

While other studies may conduct cross-system comparisons, especially in OCT, our goal is to create a manufacturer-agnostic solution that enables any ultrasound system to image ophthalmic tissues in 3D. Thus, inter-manufacturer comparisons are beyond the scope of this manuscript.

Limited Discussion on Limitations:
The discussion briefly touches on some limitations, such as patient discomfort and motion artifacts during scanning, but these points could be elaborated further. For instance, more attention could be given to the impact of motion artifacts and how they might be mitigated.
Additionally, the limitations of using different ultrasound technologies (CMUT vs. piezoelectric transducers) and their effects on image quality could be discussed in more detail.

**Response**: We appreciate the reviewer’s feedback on expanding the discussion of limitations. To clarify the impact of motion artifacts, we have added the following to the discussion: “Subjects typically required several scans to acquire usable data free from significant motion artifacts.” Our primary approach to mitigating these artifacts involved optimizing scanning speed to reduce the likelihood of subject movement during the scan. This was mainly examined in our phantom imaging section (Fig 3), where we explored the tradeoff between actuation speed and imaging quality.

While we utilized different ultrasound technologies in this study (including CMUT and piezoelectric transducers), the authors believe that a detailed analysis of the limitations, image quality, and advantages of each technology would be best suited to a dedicated study. The primary goal of this manuscript was not to quantitatively evaluate each technology but rather to demonstrate their application within a custom slit lamp-mounted instrument to actuate existing commercial ultrasound probes.

Patient Sample Size:
The paper includes in vivo imaging of a small number of subjects, which limits the generalizability of the findings. Future studies should aim to evaluate the system's performance on a larger cohort of patients with a wider variety of ocular conditions to better understand its clinical utility.

**Response**: The authors agree with Reviewer #3 that future studies should assess the system’s performance in a larger cohort of patients and across a broader range of ocular conditions to better determine its clinical utility. We have added this as a limitation in the discussion section:

“Further studies are needed to develop quantitative methods to analyze 3D models of customized US phantoms to refine accuracy in 3D model reconstruction. While the study highlights the clinical potential of the 3D ocular US device by presenting normal and pathological human eyes in vivo, drawing definitive conclusions regarding its clinical utility over traditional 2D US demands clinical investigation in larger cohorts of patients across a wider variety of ocular conditions.”

Future Research Directions:
While the paper suggests further research is needed, it would benefit from a more detailed outline of the specific next steps. For example, what improvements in the hardware or software could enhance the system? How might machine learning techniques be integrated to automate the interpretation of the 3D images?

**Response**: We thank the reviewer for this comment regarding future research directions and have added the following to our discussion section to address these concerns: “Clinically, the 3D ocular US device would benefit from a graphical user interface that would allow for more seamless acquisition and recording of data. Further studies with larger 3D imaging datasets could employ machine learning to automatically segment ocular structures of interest, detect ocular pathologies, and potentially adjust for motion and other artifacts.”
