# Supplementary material for: 3D ophthalmic ultrasonography at the slit lamp using existing ultrasound systems
Source: PLoS One. 2025 Jan 28;20(1):e0317885. doi: 10.1371/journal.pone.0317885 (PMC11774342; doi:10.1371/journal.pone.0317885)
Supplement: Attachment — Submitted filename: Response to Reviewers.docx [file pone.0317885.s004.docx]

**Response to Reviewers**

We are enclosing a revised version of our manuscript entitled “3D ophthalmic ultrasonography at the slit lamp using existing ultrasound systems”. The reviewers’ comments are in blue text. Author responses are in black text.

We would like to thank the reviewers for their comments and efforts in improving our manuscript. Our revised manuscript addresses all comments made by the reviewers. A detailed, point-by-point reply to each of the comments is listed below.

Review Comments to the Author:

Reviewer #1: All questions were clarified by the author, including the relevance of the method they are presenting.

**Response**:

We would like to thank reviewer #1 for their efforts in improving our manuscript.

Reviewer #3: The manuscript is a valuable contribution to the field and is suitable for publication once the following recommended modifications are implemented:
Quantitative Analysis:
Include at least basic quantitative performance metrics, such as resolution or noise levels, to strengthen the scientific rigor.
Probe Specifications:
Add detailed technical specifications of the probes in the Methods section for completeness and transparency.
Expanded Discussion:
Further elaborate on how motion artifacts and patient comfort issues might be mitigated in future iterations of the system.

**Response**:

We would like to thank reviewer #3 for their comments.

1. **Quantitative analysis:** To analyze noise levels from the Accutome, ABSolu, and Butterfly iQ probes using our device, we included Mean Signal (μ) and Standard Deviation (σ, Noise). The analysis was performed as follows: clear gelatin phantoms, created as described in the manuscript, were imaged using each ultrasound probe in the 3D ultrasound device. An area of the image not near any structures or borders within the phantom was sampled to isolate noise levels. These sampled images were then analyzed using ImageJ to calculate the Mean Signal and Standard Deviation.

**S1 Fig. Noise analysis of clear gelatin phantom imaging with the Accutome B-Scan Pro, ABSolu 20 MHz Annular Ring Probe, and Butterfly iQ (Ophthalmic Preset).** Mean Signal (μ) is labelled in blue with Standard Deviation (σ, Noise) as error bars.

These results indicate differing amounts of signal variability in gelatin, a tissue-like medium. We would like to again emphasize that this manuscript is not focused on quantifying each probe’s intrinsic signal to noise ratio, and the impact of a specific probe’s signal to noise ratio on 3D reconstruction of features of different sizes in real world pathology may be done in future research.

While the resolution of the ultrasound probes has been investigated previously (please also see Thomas et al., 2024 <https://pubmed.ncbi.nlm.nih.gov/38739643/>), qualitative analysis of resolution through phantom imaging was performed in this study. Additionally, we included resolution specifications for the Accutome and ABSolu from the manufacturers in the table below that will be included in the Supporting Information section.

We have added these findings in a Supporting Information section and included analysis of signal variability represented as standard deviation in the Discussion section.

2. **Probe specifications:** We compiled technical specifications for each probe based on information from the manufacturers’ websites and included the following table in the Supporting Information section for comparison:

| Feature | Accutome B-Scan Pro | ABSolu 20 MHz Annular Ring Probe | Butterfly iQ (Ophthalmic Preset) |
| --- | --- | --- | --- |
| Frequency | 12 MHz | 20 MHz | Estimated 10–20 MHz (exact not disclosed) |
| Imaging Mode | B-Mode | B-Mode | B-Mode |
| Depth of Field | 30 mm and 60 mm | 20 mm | 50 mm |
| Axial Resolution | 15 µm | 80 µm | Not explicitly stated |
| Lateral Resolution | 85 µm | 200 µm | Not explicitly stated |
| Focal Point | 22 mm | 22 mm | Not explicitly stated |
| Field of View | 60 degrees | 50 degrees | Not explicitly stated |
| Probe Type | Piezoelectric | Annular 5 rings | CMUT |
| Application Scope | Specialized for posterior segment | Specialized for posterior segment | Multi-organ use with ophthalmic preset |
| Price Range | $12,000–$18,000 | Approx. $30,000+ | $2,000–$3,000 subscription-based |

3. **Expanded discussion** **on improving motion artifacts and patient comfort:**

To further expand upon how our device could be improved for motion artifacts and patient comfort, we have added the following to our discussion:

“To minimize motion artifacts, future iterations of the device should investigate increasing scan speed, implementing real-time motion tracking and correction, enhancing patient stabilization with improved support systems, exploring redundant scanning paths, cross-referencing with other imaging modalities, and advanced post-processing techniques. To improve patient comfort, future studies with manufacturers may further optimize the probe-to-tissue interface with novel immersion-based methods and enhance corneal anesthesia and lubrication.”

We thank Reviewer #3 for their contributions to our help us improve the relevance of our manuscript.
